# Adhesive Plaster in the Fracture of Patella

**Published:** 1854-11

**Authors:** 

**Affiliations:** Surgeon to the Pennsylvania Hospital


### ON THE USE OF ADHESIVE PLASTER IN THE TREATMENT OF FRACTURE OF THE PATELLA.

BY DR. NEILL,

Surgeon to the Pennsylvania Hospital.

(*Pennsylvania Medical Examiner, January,* 1854.)

Dr. Neill's plan is to bring together the fragments of the fractured patella by means of long and broad straps of adhesive plaster (1¼ inches wide.) He carries each of these straps round the lower third of the thigh, so as to press the muscles and the detached fragment of bone towards the knee, and then, bringing it across the popliteal space, he brings it back again by carrying it round the leg, immediately below the inferior edge of the patella. Four or five of these straps are applied, each one overlapping the former one, until the coils of plaster above the knee extend down to the edge of the patella. Two cases are related, in which this plan answered very well, in conjunction with ordinary and simple means for keeping the limb extended during the treatment.

